# The Relationship between GRACE Score and Epicardial Fat Thickness in non-STEMI Patients

**DOI:** 10.5935/abc.20160024

**Published:** 2016-03

**Authors:** Ilker Gul, Mustafa Zungur, Ahmet Cagri Aykan, Teyyar Gokdeniz, Ezgi Kalaycioğlu, Turhan Turan, Engin Hatem, Faruk Boyaci

**Affiliations:** 1 Şifa University Faculty of Medicine - Department of Cardiology, Izmir - Turquia; 2 Ahi Evren Thoracic and Cardiovascular Surgery Training and Research Hospital - Department of Cardiology, Trabzon - Turquia; 3 Kafkas University Faculty of Medicine - Department of Cardiology, Kars - Turquia; 4 Erzurum Region Education and Research Hospital, Erzurum - Turquia; 5 Samsun Education and Research Hospital, Samsun - Turquia

**Keywords:** Acute Coronary Syndrome, Adipose Tissue, Myocardial Infarction, Echocardiography, Pericardium

## Abstract

**Background:**

GRACE risk score (GS) is a scoring system which has a prognostic significance in patients with non-ST segment elevation myocardial infarction (non-STEMI).

**Objective:**

The present study aimed to determine whether end-systolic or end-diastolic epicardial fat thickness (EFT) is more closely associated with high-risk non-STEMI patients according to the GS.

**Methods:**

We evaluated 207 patients who had non-STEMI beginning from October 2012 to February 2013, and 162 of them were included in the study (115 males, mean age: 66.6 ± 12.8 years). End-systolic and end-diastolic EFTs were measured with echocardiographic methods. Patients with high in-hospital GS were categorized as the H-GS group (in hospital GS > 140), while other patients were categorized as the low-to-moderate risk group (LM-GS).

**Results:**

Systolic and diastolic blood pressures of H-GS patients were lower than those of LM-GS patients, and the average heart rate was higher in this group. End-systolic EFT and end-diastolic EFT were significantly higher in the H-GS group. The echocardiographic assessment of right and left ventricles showed significantly decreased ejection fraction in both ventricles in the H-GS group. The highest correlation was found between GS and end-diastolic EFT (r = 0.438).

**Conclusion:**

End-systolic and end-diastolic EFTs were found to be increased in the H-GS group. However, end-diastolic EFT and GS had better correlation than end-systolic EFT and GS.

## Introduction

Various scoring systems have been developed for prognostic assessment of patients hospitalized with acute coronary syndromes (ACS). American Heart Association (AHA) and European Society of Cardiology (ESC) guidelines emphasize the prognostic importance of GRACE risk score (GS), and recommend it for routine use. GS can be calculated for the in-hospital period and for the six months following discharge from the hospital. In-hospital GS > 140 is considered as increased risk in terms of mortality rates. Early intervention and revascularization therapies are recommended for these patients.^1,2^

Visceral adipose tissue is known to have endocrine and metabolic activity functions. Epicardial fat (adipose) tissue is a type of visceral adipose tissue that extends along with the coronary arteries. The importance of epicardial fat thickness (EFT) has been shown by growing amount of supportive data in recent years. Increased EFT is associated with hypertension, insulin resistance and inflammatory processes such as diabetes mellitus and metabolic syndrome.^3-5^ As a result of the studies investigating the relationship between EFT and coronary artery disease (CAD), EFT was associated with severity and burden of CAD.^6-11^

EFT can be measured by means of magnetic resonance imaging (MRI) and multi-detector computed tomography (CT). However, these tests are quite expensive and not for routine use. Echocardiography is quite inexpensive, readily available and easy to perform compared to CT and MRI. For these reasons, the majority of previous studies have calculated EFT using echocardiographic methods. There is no consensus on during which phase of the cardiac cycle EFT should be measured. Although some studies in the literature suggest using end-systolic EFT measurements,^7,11,12^ a greater number of studies recommend end-diastolic EFT measurements.^5-8^

The present study aimed to determine whether end-systolic or end-diastolic EFT is more closely associated with high-risk non-ST segment elevation myocardial infarction (non-STEMI) patients according to the GS.

## Methods

This prospective observational cohort study was carried out between October 2012 and February 2013. Patients who had non-STEMI for the first time were included in the study. Patients with chronic lung disease, chronic liver disease, inflammatory rheumatic disease, muscle disease, heart failure, chronic renal failure (creatinine > 2.5 mg/dL), myocarditis, and cardiomyopathic disease were excluded from the study.

Non-STEMI was diagnosed in the presence of an accelerating pattern of or prolonged angina or recurrent episodes of angina either at rest or during minimal exertion within the last 48-72 hours and levels of troponin or creatine kinase-MB (CK-MB) above the upper limit of normal range. Biochemical parameters were evaluated in blood samples collected during hospitalization. Peak cTnI and CK-MB levels were identified during the cardiac enzyme follow-up.

The GS was calculated by means of a computer program (www.outcomes-umassmed.org/grace/acs_risk). Age, systolic blood pressure, serum creatinine level, and Killip class were determined for GS. Cardiac arrest on admission, ST-segment changes, the method of revascularization and data about the increase in cardiac enzymes were entered into the computer program as appropriate. After GS values were calculated, the patients were divided into groups as recommended by the AHA and ESC guidelines on non-STEMI.^1,2^

Patients with low-to-moderate in-hospital GS (< 140) were categorized as the low-to-moderate GRACE score (LM-GS) group (n = 70) while the patients with high in-hospital GS (> 140) were categorized as the high GRACE score (H-GS) group (n = 92).

Baseline clinical [heart rate, blood pressure, electrocardiography, laboratory biochemical parameters, angiography], echocardiographic and demographic [age, gender, height, weight] variables of the patients were recorded. The predictor (grouping) variable was the GS, and the primary outcome variable was EFT.

### Echocardiography

Echocardiographic analysis was performed in left lateral decubitus position according to the American Society of Echocardiography guidelines, within the first 24 hours in intensive care unit using the Vivid S5 cardiovascular ultrasound system (General Electric Vingmed Ultrasound, Horten, Norway). Epicardial fat was defined as an echo-free space between the outer wall of the myocardium and the visceral layer of the pericardium. The largest diameter of epicardial fat located on the right ventricular (RV) free wall was determined. EFT was measured in the parasternal long axis view at end-systole and end-diastole in three cardiac cycles. The average of three cardiac cycles was used for statistical analysis.

Left ventricular ejection fraction (LVEF) was determined using the Modified Simpson method. For a better evaluation of the RV functions, tricuspid annular plane systolic excursion (TAPSE) amount was calculated from the lateral tricuspid annulus.^13^ The right ventricular ejection fraction (RVEF) was calculated with 3D echocardiography using the disk summation method. The myocardial performance index, also known as Tei index, is a global estimate of both RV functions. Tei index is a Doppler-derived time interval index that combines both systolic and diastolic cardiac performance. The Tei index is easily derived using conventional pulsed Doppler echocardiography, as previously described by Tei et al.^14^

The present study has been designed and conducted in accordance with the Declaration of Helsinki, and the protocol has been approved by the Ethics Committee.

### Variability analysis

Intra- and inter-observer variability were assessed for the echocardiographic data obtained from a subgroup of 40 subjects. One day later, the first operator repeated the analysis to assess intra-observer variability. To assess inter-observer variability, the second operator who was blinded to the previous measurements analyzed each parameter two days later. Agreement analysis for inter- and intra-observer variability of EFT measurements revealed a high level of agreement with a mean difference of 0.26 (95% limit of agreement: -0.5 to 0.93) and an intraclass correlation coefficient of 0.918 (95% CI 0.830-0.960).

### Statistical analysis

Categorical data are presented as frequencies (percentages). Continuous variables are presented as mean ± SD. Kolmogorov-Smirnov test was used to assess whether patients' data fit normal distribution. As the data exhibited normal distribution, the independent-samples t-test was used for statistical analysis. Chi-square analysis was performed for categorical variables. Intraclass correlation coefficients and Bland-Altman analysis were used for echocardiographic measurements to assess intra- and inter-observer reproducibility, respectively. Pearson's test was used to analyze the correlation between GS and EFT. Receiver operating characteristic (ROC) analysis was performed to determine whether EFT could predict high-risk groups based on GS scores. A two-tailed p < 0.05 was considered statistically significant.

## Results

### Baseline demographics and characteristics

We evaluated 207 patients admitted to our center with non-STEMI, and 45 of them were excluded from the study (chronic lung disease, 22 patients; chronic liver disease, 2 patients; previous heart failure, 10 patients; chronic renal failure, 8 patients; myocarditis, 2 patients; and cardiomyopathic disease, 1 patient). Totally 162 patients were included in the study and 115 of them were male, and the average age was 63.9 ± 12.8 years. According to in-hospital GS, 70 patients were categorized as the LM-GS group, and 92 patients were categorized as the H-GS group. The mean age was higher in the H-GS group (73.7 ± 10.5 vs. 57.2 ± 9.1 years, p < 0.001). Mean systolic blood pressure (147.3 ± 27.7 vs. 130.3 ± 31.1 mmHg, p < 0.001) and mean diastolic blood pressure (85.5 ± 16.1 vs. 78.1 ± 19.5 mmHg, p = 0.010) values were higher in the LM-GS group. There was no difference between the groups in terms of body mass index, smoking, and the number of patients with hypertension, and diabetes mellitus. Mortality rate [3 (4.3%) vs. 13 (14.1%), p = 0.037], length of stay in intensive care unit [3.3 ± 1.2 days vs. 4.3 ± 2.9 days, p = 0.019] and total length of hospital stay [5.6 ± 2.5 days vs. 6.9 ± 3.8 days, p = 0.020] were significantly higher in the H-GS group compared to the LM-GS group (Table 1).

**Table 1:** General characteristics of patients with non-ST-elevatlon myocardial Infarction according to the in-hospital GRACE score

	**Low-to-moderate risk group (n = 70)**	**High risk group (n = 92)**	**p value**
Age, years	57.2 ± 9.1	73.7 ± 10.5	p < 0.001
Gender, male/female	56/14	59/33	p = 0.027
Systolic Blood Pressure, mmHg	147.3 ± 27.7	130.3 ± 31.1	p < 0.001
Diastolic Blood Pressure, mmHg	85.5 ± 16.1	78.1 ± 19.5	p = 0.010
Body Mass Index, kg/m^2^	28.5 ± 4.4	26.7 ± 3.7	p = 0.060
Smoking, n (%)	35 (50%)	50 (54.1%)	p = 0.583
Hypertension, n (%)	40 (57.1%)	44 (47.8%)	p = 0.299
Diabetes mellitus, n (%)	21 (30.0%)	33 (35.9%)	p = 0.376
Blood Glucose on admission, mg/dL	139.6 ± 60.3	146.5 ± 70.3	p = 0.515
Total Cholesterol, mg/dL	185.7 ± 44.9	204.2 ± 55.9	p = 0.022
LDL Cholesterol, mg/dL	133.1 ± 37.9	148.5 ± 43.9	p = 0.019
HDL Cholesterol, mg/dL	40.9 ± 15.9	39.9 ± 9.8	p = 0.627
Triglycerides, mg/dL	124.8 ± 71.4	131.3 ± 96.8	p = 0.641
C-Reactive Protein, mg/dL	1.63 ± 3.4	2.68 ± 4.38	p = 0.013
Creatinine, mg/dL	0.84 ± 0.2	1.18 ± 0.57	p < 0.001
Troponin-I, ng/mL	31.6 ± 30.0	31.1 ± 28.1	p = 0.951
LV Ejection Fraction, %	47.8 ± 10.2	43.3 ± 10.5	p = 0.008
Interventricular septum, mm	10.1 ± 1.2	10.1 ± 1.36	p = 0.726
Posterior Wall, mm	9.8 ± 1.12	9.9 ± 1.26	p = 0.761
LV Mass, g	181.5 ± 44.2	198.7 ± 49.3	p = 0.019
LV Mass Index, g/m^2^	94.3 ± 22.4	109.4 ± 28.4	p = 0.002
Epicardial fat thickness (systole), cm	0.72 ± 0.17	0.81 ± 0.12	p < 0.001
Epicardial fat thickness (diastole), cm	0.46 ± 0.13	0.54 ± 0.10	p < 0.001
SPAP, mmHg	25.1 ± 7.5	30.7 ± 8.7	p < 0.001
TAPSE, mm	20.9 ± 4.1	19.4 ± 3.6	p = 0.017
RV Ejection Fraction, %	53.1 ± 5.3	50.1 ± 5.4	p = 0.001
Tei Index	0.35 ± 0.09	0.44 ± 0.11	p < 0.001
SYNTAX score	20.6 ± 10.1	21.2 ± 9.9	p = 0.625
LAD lesion, n (%)	29 (41.4%)	35 (38.0%)	p = 0.744
Cx lesion, n (%)	17 (24.3%)	16 (17.4%)	p = 0.280
RCA lesion, n (%)	24 (34.3%)	41 (44.6%)	p = 0.149
Mortality, n (%)	3 (4.3%)	13 (14.1%)	p = 0.037
Duration in intensive care unit, days	3.3 ± 1.2	4.3 ± 2.9	p = 0.019
Total length of hospital stay, days	5.6 ± 2.5	6.9 ± 3.8	p = 0.020

Data are presented as mean±SD or number (percentage) *Chi-square and independent sample t-test LV: Left ventricular; RV: right ventricular; Cx: circumflex artery; HDL: high density lipoprotein; LAD: left anterior descending artery; LDL: low-density lipoprotein; RCA: right coronary artery; SPAP: systolic pulmonary artery pressure; TAPSE: tricuspid annular plane systolic excursion.

### Laboratory findings

Evaluation of the laboratory data revealed no difference between the average values of postprandial blood glucose, HDL-cholesterol, triglycerides, and cTn-I levels. The mean values of total cholesterol (204.2 ± 55.9 vs. 185.7 ± 44.9 mg/dL, p = 0.022), LDL-cholesterol (148.5±43.9 vs. 133.1 ± 37.9 mg/dL, p = 0.019), C-reactive protein (2.68 ± 4.38 vs. 1.63 ± 3.4 mg/L, p = 0.013) and creatinine (1.18 ± 0.57 vs. 0.84 ± 0.20 mg/dL, p < 0.001) were higher in the H-GS group compared to the LM-GS group (Table 1).

### Echocardiography and coronary angiography

There was no difference between the groups regarding interventricular septum and posterior wall thickness. EF was lower in the H-GS group (43.3% ± 10.5 vs. 47.8% ± 10.2, p = 0.008). End-systolic EFT (0.81 ± 0.12 vs. 0.72 ± 0.17 cm, p < 0.001), end-diastolic EFT (0.54 ± 0.10 vs. 0.46 ± 0.13 cm, p < 0.001) and systolic pulmonary artery pressure (SPAP) (30.7 ± 8.7 vs. 25.1 ± 7.5 mmHg, p < 0.001) were higher in the H-GS group. RVEF, one of the indicators of RV systolic function (50.1% ± 5.4 vs. 53.1% ± 5.3, p = 0.001) and TAPSE (19.4 ± 3.6 vs. 20.9 ± 4.1, p = 0.017) were lower in the H-GS group than in the LM-GS group (Table 1). Tei index was significantly higher in the H-GS group (0.48 ± 0.09 vs. 0.39 ± 0.08, p < 0.001).

Evaluation of the patients' angiograms showed no significant difference between left anterior descending (LAD), circumflex (Cx) and right coronary artery (RCA) involvements (Table 1).

### Correlation and ROC analysis

**Figure 1:**
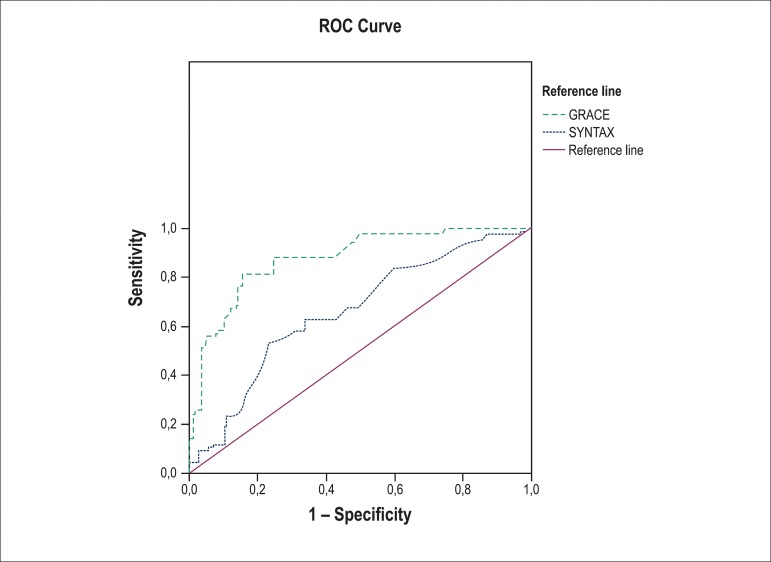
Receiver operating characteristic (ROC) curve.The end-systolic epicardial fat thickness cut-off value which predicts the H-GS group was calculated as 0.765 cm (AUC: 0.699; 95% CI: 0.613-0.785; sensitivity: 67%; specificity: 64%). End-diastolic epicardial fat thickness cut-off value was found to be 0.468 cm for the H-GS group (AUC: 0.709; 95% CI: 0.621-0.797; sensitivity: 68%; specificity: 72%). (AUC: Area Under the Curve; CI: Confidence Interval; H-GS: High GRACE score).

Based on in-hospital GS, the ROC analysis was performed to calculate the EFT for prediction of patients with high-risk. The end-systolic EFT cut-off value which predicts the H-GS group was calculated as 0.765 cm (AUC: 0.699; 95% CI: 0.613-0.785; sensitivity, 67%; specificity, 64%), and the end-diastolic EFT cut-off value was found to be 0.468 cm for the H-GS group (AUC: 0.709; 95% CI: 0.621-0.797; sensitivity, 68%; specificity, 72%) (Table 2, Figure 1).

**Table 2:** According to in-hospital GRACE scores (GS), the receiver operating characteristic (ROC) analysis was performed to calculate the epicardial fat thickness (EFT) for the prediction of patients at high risk

	**Cut-off (cm)**	**Sensitivity**	**Specificity**	**AUC**	**95% Confidence Interval**
EFT end-systole / GS	0.765	67%	64%	0.699	0.613-0.785
EFT end-diastole / GS	0.468	68%	72%	0.709	0.621-0.797

Receiver operating characteristic (ROC) analysis AUC: area under the curve.

The correlation coefficient (r) of in-hospital GS and end-systolic EFT was found as 0.387, while the correlation coefficient (r) of in-hospital GS and end-diastolic EFT was found as 0.438 (Table 3).

**Table 3:** Correlation table for the in-hospital GRACE score (GS), SYNTAX score and epicardial fat thickness (EFT) measurements

	**Correlation coefficient (r)**	**p value**
End-systolic EFT - GS	r = 0.387	< 0.001
End-diastolic EFT - GS	r = 0.438	< 0.001
End-systolic EFT - SYNTAX score	r = 0.285	0.009
End-diastolic EFT - SYNTAX score	r = 0.292	0.002

Pearson’s correlation analysis

## Discussion

In this study, we aimed to show the relationship between the GS and EFT in patients admitted to our center with a diagnosis of non-STEMI. Our study demonstrated that EFT is associated with increased GS, and statistical analysis suggests that it would be more convenient to measure EFT at the end of diastole.

GRACE scoring system was initially used for the purpose of following a prognostic assessment of patients with ACS after the multinational Global Registry of Acute Coronary Events (GRACE) study.^15,16^ Detection of a high GS calculated with the help of variables is associated with poor prognosis. Currently, the ACS guidelines recommend its use for risk evaluation. The AHA and ESC ACS diagnosis and treatment guidelines provide suggestions on how to interpret GS. According to these recommendations, in-hospital GS > 140 is an indicator of high morbidity and mortality. These guidelines recommend early revascularization for patients at high risk.^1,2^ In our study, a higher mortality rate was found (p = 0.001) in the H-GS group with GS > 140. Apart from mortality rates, echocardiographic parameters decreased in the H-GS group. The LVEF values of the H-GS group were lower than those of the LM-GS group. The RVEF and TAPSE, which are markers of RV systolic functions, were decreased in the H-GS group. The Tei index and SPAP were increased in the H-GS group.

Epicardial fat is a type of visceral adipose tissue that extends along coronary arteries in atrioventricular and interventricular grooves. Myocardium and epicardial fat tissue are in direct contact, and the blood supply for these tissues is provided by the same coronary system.^17^ Therefore, acting as an endocrine organ, epicardial fat tissue can directly and indirectly affect coronary arteries and cardiac structures. Epicardial fat tissue can produce several cytokines that stimulate angiogenesis, inflammation, oxidative stress and atherosclerosis. A number of recent clinical studies have been conducted to investigate the relationship between EFT and cardiovascular diseases. As a result of these studies, increased EFT has been associated with an increased risk of cardiovascular disease.^5-7,11,18-22^ EFT is increased in patients with inflammation, advanced age, obesity, hypercholesterolemia, hypertension, atrial fibrillation, diabetes mellitus and metabolic syndrome.^3,4,10,23-27^ In our study, there was no significant difference between the groups in terms of blood glucose and body mass index. The number of patients diagnosed with metabolic syndrome, diabetes and hypertension was similar between groups (Table 1).

There are several recommendations on how to measure EFT. The RV free wall is preferred as the measurement site, and measurements for the calculation of EFT are generally performed at the end of diastole.^5,8^ However, some publications suggest that EFT can also be measured at the end of systole.^(^7^,^^11^^,^^12^^)^ It is recommended that EFT should be measured at the end of systole during the cardiac cycle as it is compressed during diastole. In the present study, average EFT values were obtained by employing at least three different measurements for end of systole and end of diastole, and the average EFT values were higher in the H-GS group compared to the LM-GS group (p < 0.001) (Table 1). A correlation was observed between in-hospital GS and EFT (in-hospital GS and end-systolic EFT, r = 0.387; end-diastolic, r = 0.438). Evaluation of ROC analyses demonstrated that end-diastolic EFT provided a better prediction of high GS compared to end-systolic EFT (Table 2, Figure 1).

### Study limitations

The main limitation of the present study is the relatively small sample size. Secondly, echocardiography is less accurate than other radiological techniques for evaluating epicardial adipose tissue, although a good correlation with MRI has been reported. Moreover, a moderate correlation has been reported with the epicardial fat volume assessed with cardiovascular MRI. Despite the low level of accuracy, echocardiographic measurement of EFT offers greater feasibility compared to MRI.

## Conclusions

Previous studies have shown increased EFT in various conditions and pathologies that may impair cardiac functions. In our study mortality and morbidity rates were increased in H-GS group. The GS showed a positive correlation with end-systolic EFT and end-diastolic EFT. Statistical evaluations demonstrated a better correlation between GS and end-diastolic EFT rather than end-systolic EFT.

In conclusion, calculating the end-diastolic EFT may provide useful information in patients with high GS during the echocardiographic evaluation of non-STEMI.
